# Does the Composition of Gut Microbiota Affect Chronic Kidney Disease? Molecular Mechanisms Contributed to Decreasing Glomerular Filtration Rate

**DOI:** 10.3390/ijms251910429

**Published:** 2024-09-27

**Authors:** Ewelina Młynarska, Emilian Budny, Maciej Saar, Ewa Wojtanowska, Justyna Jankowska, Szymon Marciszuk, Marcin Mazur, Jacek Rysz, Beata Franczyk

**Affiliations:** 1 Department of Nephrocardiology, Medical University of Lodz, ul. Zeromskiego 113, 90-549 Lodz, Polandmarcin.mazur.publ@gmail.com (M.M.);; 2 Department of Nephrology, Hypertension and Family Medicine, Medical University of Lodz, ul. Zeromskiego 113, 90-549 Lodz, Poland

**Keywords:** gut microbiota, microbiome, chronic kidney disease (CKD), uremic toxins, probiotics, prebiotics

## Abstract

Chronic kidney disease (CKD) is a very prevalent and insidious disease, particularly with initially poorly manifested symptoms that progressively culminate in the manifestation of an advanced stage of the condition. The gradual impairment of kidney function, particularly decreased filtration capacity, results in the retention of uremic toxins and affects numerous molecular mechanisms within the body. The dysbiotic intestinal microbiome plays a crucial role in the accumulation of protein-bound uremic toxins such as p-cresol (pC), indoxyl sulfate (IS), and p-cresyl sulfate (p-CS) through the ongoing fermentation process. The described phenomenon leads to an elevated level of oxidative stress and inflammation, subsequently resulting in tissue damage and complications, particularly an increase in cardiovascular risk, representing the predominant cause of mortality in chronic kidney disease (CKD). Therefore, exploring methods to reduce uremic toxins is currently a pivotal therapeutic strategy aimed at reducing the risk of organ damage in patients with chronic kidney disease (CKD). This review aims to summarize recent discoveries on modifying the composition of the intestinal microbiota through the introduction of special probiotic and synbiotic supplements for CKD therapy. The potential to connect the gut microbiota with CKD opens the possibility for further extensive research in this area, which could lead to the incorporation of synbiotics and probiotics into the fundamental treatment and prevention of CKD.

## 1. Introduction

Chronic kidney disease (CKD) is a significant global health concern, with a high prevalence, poorly expressed symptoms in the early stages of the disease, and co-morbidity with other disease entities. Approximately 13.4% of the population is affected by CKD, with the majority (10.6%) in the late stages of the disease (stages 3–5) and 0.1% in stage 5 [1]. However, the percentage provided above may be inaccurate as the population suffering from CKD in stages 1–2 may not be aware of their condition, which could lead to its under detection and warping of the data above [2]. The number of patients at stage 5 is low because people with chronic kidney disease (CKD) are five to ten times more likely to die prematurely than to develop kidney failure [3].

Chronic kidney disease is a progressive and irreversible condition that results in the gradual loss of nephrons, leading to an overload of the remaining nephrons through hyperfiltration. This, in turn, causes glomerular hypertrophy, followed by sclerosis and fibrosis of the interstitium [4]. The progressive kidney damage and reduced filtration result in the accumulation of several substances called uremic toxins in the body. It is hypothesized that there is a correlation between the presence of uremic toxins, the development of inflammation and immune dysfunction, and the onset of vascular disease. In addition, uremic toxins have been linked to abnormalities in platelet function, altered drug metabolism, dysbiosis in the gut (including increased bacterial translocation), and the advancement of CKD [3,5,6]. Moreover, the 1-alpha-hydroxylation of vitamin D, which occurs in the kidneys, is also impaired, which is the cause of hypocalcemia and secondary hyperparathyroidism [7]. Additionally, in CKD, anemia is caused primarily by reduced erythropoietin production by the kidneys. Damaged kidneys cannot maintain normal blood pH, volume, and adequate electrolyte composition [8].

Furthermore, research findings confirm that patients with chronic kidney disease (CKD) exhibit an altered composition of gut microbiota when correlated to healthy controls (HC) [9,10,11,12]. In comparison to the control group at the phylum level, CKD patients demonstrated an increased presence of Proteobacteria and Bacteriodetes while exhibiting reduced levels of Actinobacteria and Firmicutes. In CKD patients, at the genus level, a higher quantity of *Bacteroides*, *Prevotella*, and *Klebsiella* were detected, while a lowered count of *Bifidobacterium*, *Collinsella*, *Dorea*, *Oscillospira*, and *Akkermansia* were observed in contrast with those of the HC group. In addition, the study demonstrated a reduction in the diversity of the gut microbiota in patients with CKD. These findings suggest a dysbiosis in the gut microbiome of CKD patients [12].

The most common causes of chronic kidney disease are type 2 diabetes and arterial hypertension (AH) [4]. Furthermore, the composition of the gut microbiota and its metabolites could be associated with the onset and progression of each of these diseases [13,14].

Recently, CKD has been gaining popularity amongst scientists and clinicians as their recommended treatment and care are still not up to par [15,16]. This fact emphasizes the demand for further investigation into other treatment options [16]. The connection between CKD and gut microbiota suggests the usefulness of including probiotics and synbiotics in the therapy. The inclusion of such supplements is said to help regulate the balance of the intestinal flora, which may lead to suppression of the progression of CKD [16,17,18,19]. As it has been proven in several animal studies, the supplementation of *Lactobacillus* slows the progression of CKD and delays the occurrence of renal failure by altering short-chain fatty acid and nicotinamide metabolism [16,20]. After introducing probiotics to the treatment, the tendency to reduce the levels of tumor necrosis factor-α (TNF-α), interleukin (IL)-6, IL-18, and endotoxin in serum occurred [16,21].

The objective of this review is to describe the role of gut microbiota in renal failure, taking into account the molecular processes involved and new insights into the treatment of chronic kidney disease (CKD).

## 2. Review Methodology

The comprehensive analysis was performed on papers published between 2004 and 2023. Emphasis was placed on articles pertaining to microbiota composition and probiotics, which were published between 2017 and 2024.

The materials for the review were identified through the use of the following keywords: “gut microbiota”, “chronic kidney disease”, “uremic toxins”, “probiotics”, and “prebiotics”. The analysis included both English-language and non-English-language publications.

The study population analyzed in the included papers included individuals with chronic kidney disease (CKD) and end-stage renal disease (ESRD), as well as matched control groups. The objective was to analyze the results of changes in the composition of the gut microbiota occurring during CKD and ESRD, including the molecular mechanisms that may influence the development and progression of these diseases and new insights into the use of probiotics and prebiotics in the treatment of kidney diseases.

## 3. What Is Renal Failure?

### 3.1. Definition, Types

Chronic kidney disease (CKD) is characterized by a gradual and irreversible progression, with a relatively insidious onset. A significant feature of the disease is the increased risk of complications, with death primarily associated with vascular diseases.

**Figure 1:**
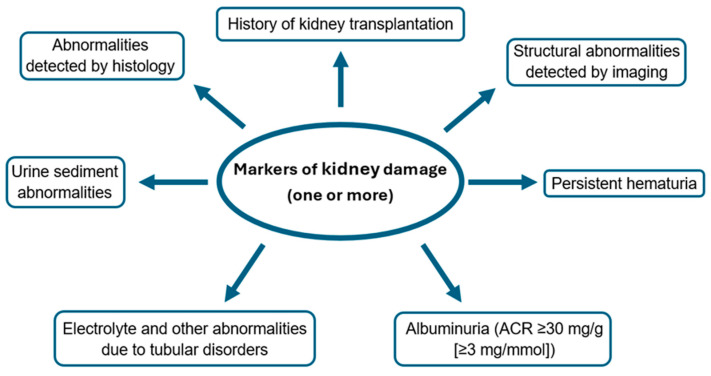
Definition of chronic kidney disease based on markers of kidney damage [22]. ACR—albumin-to-creatinine ratio.

According to Kidney Disease: Improving Global Outcomes (KDIGO), CKD is currently defined as a decreased glomerular filtration rate (GFR) <60 mL/min per 1.73 m^2^ (GFR categories G3a–G5) present for a minimum of 3 months or other abnormalities of kidney structure or function in the form of kidney damage with implications for health, present for a minimum of 3 months [22]. The abnormalities are illustrated in Figure 1.

CKD is classified into five stages based on GFR (G1–G5) and three categories based on albuminuria (A1–A3). The divisions are presented in Table 1 and Table 2.

### 3.2. Etiology and Causes

The human kidney contains approximately one million nephrons. Following the completion of organogenesis, the body is unable to produce additional nephrons [8,23]. During growth, nephrons also increase in size to align with the body’s evolving requirements. However, in situations of prolonged or sustained weight gain (e.g., during pregnancy or obesity), nephron hypertrophy (mainly involving increased dimensions of the glomerular tuft, Bowman’s capsule, and proximal tubule) occurs as a compensatory mechanism [8]. The same effect can occur with the loss of healthy nephrons (injury or donation of one kidney), i.e., hypertrophy of the remaining nephrons.

The loss of nephrons results in an increase in intraglomerular pressure, which in turn causes the glomerular walls to stretch, damaging the glomerular cells [24]. Additionally, the appearance of large pores is observed, allowing for increased filtration of plasma proteins [25,26]. Mechanical stress may also increase angiotensin II (AngII) production and angiotensin type 1 receptor expression in podocytes, and AngII may directly impair glomerular barrier screening function, probably through inhibited nephrin expression, independent of its hemodynamic effects [26,27,28]. Proteinuria is thought to lead to structural and functional damage to the nephron. Furthermore, the loading of podocytes with protein results in the release of transforming growth factor β (TGF-β), which in turn leads to the differentiation of mesangial cells into myofibroblasts [26,29,30,31]. The increased protein concentration in the tubules causes the release of chemokines, cytokines, vasoactive molecules, and growth factors by the tubule cells, which in turn leads to interstitial fibrosis through the accumulation of inflammatory cells, collagen, fibronectin, and other components [32,33]. Moreover, complement factors accumulate, exerting cytotoxic, pro-inflammatory, and fibrinogenic effects [30]. Additionally, the inflammatory process is driven by tissue damage induced by protein movement, resulting in the production of reactive oxygen species and the endoplasmic reticulum stress response [34]. Through this mechanism, oxidative modification of membrane lipids, proteins, and DNA and induction of cell death occur, resulting in tissue inflammation and local recruitment of macrophages and lymphocytes [33].

The unfavorable microenvironment is thought to promote the mesenchymal transformation of differentiated epithelial cells, possibly also endothelial cells and podocytes, which in turn may further exacerbate proteinuria and glomerular sclerosis [26,35].

Furthermore, following the damage, the process of epithelial–mesenchymal transition (EMT) is initiated in proximal tubule epithelial cells (TECs). This is regulated by E-cadherin SNAI1 and GSK3β, which is inhibited by phosphorylation of ERK and p90RSK [35,36,37,38,39,40]. The EMT process occurring in TECs has been referred to as the “failed-repair proximal tubule cell” state, often named partial EMT (pEMT) [40]. The reason for the occurrence of scarring rather than regeneration in the kidney is the prevalence of pEMT over TEC proliferation [41]. Studies of interleukin 11 (IL-11) in a mouse model have demonstrated that it also stimulates epithelial–mesenchymal transition (EMT) of epithelial cells [40,42,43]. In a mouse model of CKD, A. A. Widjaja et al. observed that the diseased mice`s kidney weight was reduced by 67%. After the IL-11 neutralizing treatment (X203) was admitted, roughly 50% of the lost kidney mass was regained, while kidney size remained unaltered in the control group. At the same time, the collagen content of the kidneys and the degree of histological fibrosis exhibited a gradual decline throughout the course of X203 therapy. The researchers observed that anti-IL11 treatment in chronic kidney disease promoted TEC regeneration, reversed fibrosis and the pEMT phenotype, and enhanced renal function [40].

### 3.3. Standard Treatment

The treatment of chronic kidney disease includes identification and treatment of the underlying cause, inhibition of disease progression, prevention and treatment of complications, treatment of comorbidities, prevention of cardiovascular disease, preparation for renal replacement therapy, and renal replacement therapy.

#### 3.3.1. Treatment of Hypertension

It is recommended that the presence and severity of albuminuria be assessed prior to initiating treatment. The use of an angiotensin-converting enzyme inhibitor (ACE-I) or an angiotensin II receptor blocker (ARB) is recommended in adults with diabetes and a urinary albumin-to-creatinine ratio (ACR) above 300 mg/24 h [44,45,46,47]. Due to the high risk of hyperkalemia and acute kidney injury, it is advisable to avoid dual therapy with ACE-I and ARB. In certain instances, the use of aldosterone receptor antagonists may be considered [44,48].

#### 3.3.2. Treatment of Diabetes Mellitus

Glycemic control represents a crucial element in the management of CKD, with evidence suggesting that achieving a target hemoglobin A1c of approximately 7.0% can delay the progression of the disease [44,45,49,50,51]. This is in line with the recommendations set forth by the majority of clinical guidelines. During therapy, drugs that are mainly removed by the kidneys should be avoided, and dose reductions or discontinuation may be required for drugs metabolized by the liver and/or partially by the kidneys, especially when eGFR is <30 mL/min/1.73 m^2^ [44,45,49]. In patients with markedly increased albuminuria, it is advisable to discontinue SGLT-2 inhibitors, if possible.

#### 3.3.3. Cardiovascular Disease Risk Reduction

The prevalence of cardiovascular disease in individuals with chronic kidney disease (CKD) is considerably higher than in those without CKD. Furthermore, individuals with CKD exhibit a more unfavorable cardiovascular prognosis [44]. Consequently, reducing cardiovascular risk is a crucial aspect of CKD treatment. It is recommended that patients aged 50 years or older with CKD should be treated with a low- or moderate-dose statin, regardless of their low-density lipoprotein cholesterol level [52,53]. It is recommended that patients should be encouraged to stop smoking [44]. Kidney Disease: Improving Global Outcomes (KDIGO) recommends a target systolic and diastolic blood pressure of less than 140 mm Hg and less than 90 mm Hg, respectively, among adults with CKD based on expert opinion [22,54].

Furthermore, patients with chronic kidney disease (CKD) should avoid nephrotoxins, which include non-steroidal anti-inflammatory drugs (NSAIDs), phosphate-based intestinal preparations, and proton pump inhibitors [44]. A comprehensive list is beyond the scope of this review.

### 3.4. Complications

The progressive reduction in the number of nephrons results in the disruption of numerous vital processes in which the kidneys are involved. The functionality of a multitude of systems could be impaired, leading to complications such as anemia, bone mineral disorders (MBD associated with vitamin D deficiency, hyperparathyroidism, hyperkalemia, and hyperphosphatemia), hypertension, increased effective circulating fluid volume, hyperuricemia, and dyslipidemia [8].

Among the complications, cardiovascular disease is of particular significance, being the leading cause of death in CKD patients worldwide. This is influenced by dyslipidemia, arterial hypertension, and hyperuricemia [55].

## 4. Molecular Basis of Renal Failure

Molecular reactions and mechanisms have the potential to regulate a multitude of processes within the human body, including those that may contribute to the development of disease. Chronic kidney disease is characterized by the destruction of the structural and functional units of the kidney, which results in an irreversible reduction in renal function. This is caused by a series of pathogenic mechanisms that target and damage the kidneys [56]. In response to relevant stimuli, inflammation is considered to be a host defense mechanism against pathogens that can generate pro-inflammatory cytokines to activate innate immunity. Moreover, the triggers of renal inflammation include bacterial and viral infections, lipid metabolism, high glucose levels, and ischemia–reperfusion injury (IRI) [57]. The inflammatory response, characterized by leukocyte infiltration and tubular cell death, is a consequence of renal (IRI). The underlying mechanisms include necroptosis and ferroptosis, which are responsible for initiating the inflammatory cascade and subsequent tissue damage [58].

### 4.1. Programmed Cell Death

**Figure 2:**
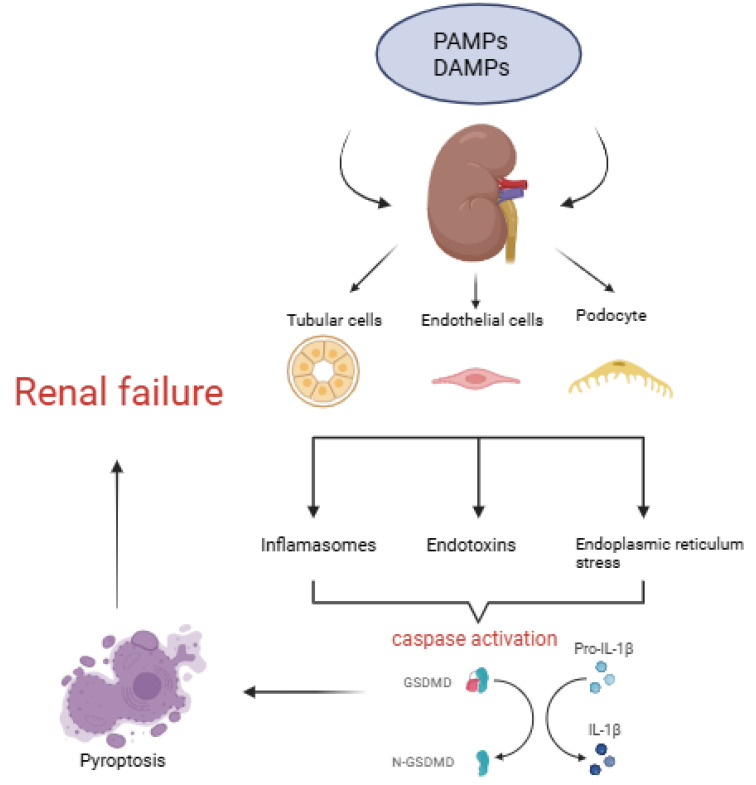
Two distinct molecular mechanisms, pathogen-associated molecular patterns (PAMPs) and damage-associated molecular patterns (DAMPs), are responsible for activating inflammatory bodies [66]. These factors induce cellular stress, which subsequently activates the inflammasome [67]. The inflammasome activates caspase-1, which in turn induces the cleavage and release of the proinflammatory cytokines IL-1β and IL-18 [68]. Cleaved caspase-1 has been established as a direct activator of GSDMD [63]. Upon cleavage of GSDMD into GSDMD-NT, the resulting fragments migrate to the membrane, where they aggregate and perforate the membrane, thereby releasing inflammatory factors such as IL-1β and IL-18, which lead to pyroptosis [69]. Several common kidney diseases have the potential to progress to end-stage renal disease (ESRD), including acute kidney injury, diabetic kidney diseases, renal fibrosis, and kidney inflammation. It has been established that all forms of kidney disease are caused by a certain level of inflammatory response. Pyroptosis, which is regulated by a variety of inflammatory bodies, plays a significant role in the progression of kidney disease [66]. PAMPs—pathogen-associated molecular patterns. DAMPs—damage-associated molecular patterns. IL—Interleukin. GSDMD—Gasdermin D.

Necroptosis could initiate the spread of cell death through ferroptosis [59]. Necroptosis to ferroptosis may be achieved by phosphatidylethanolamine-binding protein 1 and 15-lipoxygenase [60]. Due to this fact, the combined small-molecule inhibitor Necrostatin-1f, which has a strong inhibitory effect on necroptosis and a weak inhibitory effect on ferroptosis, was developed based on the relationship between necroptosis and ferroptosis [61]. Pyroptosis is a form of programmed cell death that is induced by the activation of the membrane-targeting, pore-forming gasdermin protein family (GSDM). Moreover, pyroptosis is dependent on the activity of caspases-1/4/5/11 in the context of gasdermin D (GSDMD)-mediated pyroptosis, as well as caspases-3 in the presence of gasdermin E (GSDME)-mediated pyroptosis [62]. The sequence of events leading to the activation of pyroptosis and subsequent renal failure is illustrated in Figure 2. Furthermore, it was initially characterized by the loss of membrane integrity and the secretion of cytokines such as IL-1β. Due to this fact, it was initially described as a caspase 1- and inflammasome-dependent pathway, whereby cells undergo regulated cell death in response to the activation of the NLRP3 inflammasome [63]. What is more, pyroptosis may also be associated with ferroptosis [64]. ROS-Tom20-Caspase3-GSDME-signaling pathway: iron ions and reactive oxygen species (ROS)-inducing drugs induce pyroptosis [65]. Pyroptosis is putatively implicated in the pathogenesis of kidney diseases through two distinct pathways: the caspase-1-mediated canonical pathway and the caspase-4/5/11-mediated noncanonical pathway [66].

### 4.2. The Co-Occurrence of Obesity and Renal Failure

A previous meta-analysis indicated a positive correlation between increased body mass index (BMI) and the risk of developing kidney disease (KD). This suggests that obesity significantly increases the risk of KD in the population [70]. Therefore, it has been documented that obesity-related glomerulopathy (ORG) constitutes a form of chronic kidney disease (CKD) [71]. The adverse effects of lipotoxicity on the progression of ketogenic diets among patients with acute kidney injury (AKI) and chronic kidney disease, including diabetic nephropathy (DN), ORG, and polycystic kidney disease (PKD), have been well documented [72,73]. The de novo formation of lipids is contingent upon an equilibrium between the acquisition of lipids and their subsequent disposal. This equilibrium is regulated due to three major pathways: lipid uptake, lipid synthesis, fatty acid oxidation (FAO), and lipid export. This section will examine the molecular mechanisms that regulate lipid homeostasis in KD [74]. In the kidney, the expression of cluster of differentiation 36 (CD36) can be observed in distal tubular epithelial cells (TECs), mesangial cells, podocytes, microvascular endothelial cells, and interstitial macrophages. However, the expression of CD36 in proximal tubule cells (PTCs) appears to be inconsistent [75,76,77]. Furthermore, overexpression of CD36 has been demonstrated to result in increased renal tubular damage and renal fibrosis in folic acid-treated mice compared to wild-type controls. However, there was no significant difference in renal inflammation observed [78]. In addition to the well-known inflammatory factors, recent studies have shown that the collagen type I (Col I)-mediated discoidin domain receptor 1 (DDR1) activation induces CD36-mediated podocyte lipotoxic injury, which was previously unrecognized [79].

### 4.3. Uremic Toxins

End-stage renal disease is inextricably linked to the accumulation of toxic metabolites in the blood and other metabolic compartments. This accumulation has been postulated to be associated with an increased generation of toxins from a dysbiotic microbiome, which is accompanied by a reduction in their elimination by impaired kidneys [80]. The loss of kidney function is associated with the development of intestinal dysbiosis, which is characterized by the secretion of urea into the gastrointestinal tract and the subsequent hydrolysis of urea into carbon dioxide and ammonia by certain gut microbes [81]. Patients with chronic kidney disease exhibit a diminished excretion of uremic toxins, which are hazardous metabolites, due to impaired functioning of the glomerulus and proximal tubules [82]. The presence of chronic kidney disease may be accompanied by the development of intestinal inflammation and epithelial barrier impairment. This can result in accelerated translocation of bacterial-derived uremic toxins from the gut into the systemic circulation, leading to increased oxidative stress and injury to the kidney, cardiovascular, and endocrine systems [80]. It seems that the prevalence of bacteria with fermentative activity may lead to the release and accumulation in the gut and in the blood of several substances, such as p-cresol (p-C), indoxyl sulfate (IS), and p-cresyl sulfate (p-CS) [83,84].

IS is a protein-bound uremic toxin that is derived from indole, a metabolite produced by the gut microbiota through the degradation of tryptophan. It has been demonstrated that IS causes intracellular oxidative stress and influences transcription via the aryl hydrocarbon receptor [85]. The presence of indoxyl sulfate has been demonstrated to induce pro-inflammatory responses in endothelial cells [86]. In tubular epithelial cells, indoxyl sulfate is excreted from the blood into the urine via organic anion transporters (OATs) and organic anion transporting polypeptides (OATPs), which are encoded by genes in the SLCO superfamily [87,88]. IS has been observed to induce the expression of intercellular adhesion molecule 1 (ICAM-1) in the kidney, which has been demonstrated to enhance monocyte infiltration. Additionally, it has been shown to stimulate the production of monocyte chemoattractant protein (MCP-1), a chemokine that plays a crucial role in macrophage recruitment and activation [89,90]. Furthermore, macrophage induction is a consequence of the acceleration of the aryl hydrocarbon-NF-κB/MAPK receptor cascades [91]. The transcription factor NF-κB plays a pivotal role in the pathological effects of IS in the kidneys. In human proximal tubule cells (HK-2), it has been observed that the activation of NF-κB by IS inhibits cell proliferation, induces and accelerates aging through the induction of p53, and promotes fibrosis through the induction of TGF-β1 and plasminogen activator inhibitor-1 (PAI-1) expression [92]. Furthermore, it was postulated that p53 induction may also be involved in renal fibrosis through the stimulation of TGF-β1 expression, which in turn activates the Smad3 pathway [93]. The remaining two uremic toxins, p-C and p-CS are produced as metabolites of tyrosine and phenylalanine. These are directly produced by intestinal bacteria when there is dysbiosis [94]. Furthermore, P-C has been demonstrated to induce vascular damage and genotoxicity in enterocytes [95]. It has been suggested that also p-CS may contribute to immune dysfunction by suppressing the activity of macrophages, which are pivotal in the immune system. Additionally, p-CS has been linked to the progression of renal injury and has also been demonstrated to increase ROS production and induce immunosuppression [83].

## 5. Association between Chronic Kidney Disease and Gut Microbiota

### 5.1. Basic Microbiological Information and Molecular Aspects

The influence of the gut microbiota on the human body has been a subject of scientific investigation for a considerable period [13].

Scientists show that the composition of gut microbiota could be divided into two distinct enterotypes. The first enterotype was characterized by a higher abundance of *Prevotella*, whereas the second enterotype exhibited a higher prevalence of *Bacterioides* [96]. However, some scientists have proposed a third enterotype, which is dominated by *Ruminococcus* [97]. The findings of Jiang et al. show that at the end stage of renal disease (ESRD), the total amount of gut bacteria may be reduced. Furthermore, the authors suggest that there is a possibility of a higher prevalence of *Prevotella* in the healthy group, whereas Bacteroides could be present in greater quantities in patients with ESRD [97].

The scientific literature indicates that the gut microbiota may be responsible for the generation of metabolic products, including gut-derived uremic toxins such as α-phenylacetyl-l-glutamine, 5-hydroxyindole, indoxyl glucuronide, p-cresyl sulfate (PCS), and indoxyl sulfate (IS) [98]. Of these, p-cresyl sulfate and indoxyl sulfate have been the subject of extensive study to date, which has demonstrated that they may be elevated in patients with kidney diseases [99]. Moreover, a higher presence of bacteria is correlated with higher IS and PCS levels, as evidenced by the findings of studies that have examined the relationship between the two variables. *Bacteroides* and *Blautia* appear to be associated with a high IS level, while *Enterococcus*, *Akkermansia*, *Dialister*, and *Ruminococcus* are linked to a higher PCS level [13]. Furthermore, these metabolites may exert a deleterious effect on renal tubular cells and endothelium, which could lead to their damage [84,100]. Therefore, the accumulation of uremic toxins may contribute to the progression of chronic kidney disease [84]. The possible link between gut-derived uremic toxins was also noted by S.C. Guldris et al. The authors observed clinical consequences in the form of progression of CKD due to increased inflammation and oxidative stress [101].

### 5.2. Microbiological Assessment and Used Technical Methods

The composition of the microbiota was identified from fecal samples in the analyzed papers. Bacterial DNA was isolated from the samples after purification from human DNA and amplified by a PCR reaction. Subsequently, 16S rRNA gene sequencing or metagenome shotgun sequencing was performed, followed by metagenetic analysis and gene mapping to identify specific species.

### 5.3. Quantitative and Qualitative Gut Microbiota Analysis in Patients with CKD and ESRD Compared to the Healthy Population

Some bacteria may be less abundant in chronic kidney disease. This correlation is illustrated in Table 3. Wang et al. suggested that there is a probability of lower quantities of the Clostridiaceae family without specifying a particular type of genus [11]. Moreover, greater presence of *Roseburia* and *Faecalibacterium* in the population without chronic kidney disease has been noticed in many scientific research studies [11,97,102,103,104,105]. Furthermore, a lower capacity of *Eubacterium* and *Ruminococcus* could be non-linearly correlated with a decrease in glomerular filtration rate (GFR) [11,104,105]. Studies have reported that *Bacteroides*, *Prevotella*, *Blautia*, and *Lactobacillus* could decrease in the population with CKD in comparison to the healthy population [97,102,106]. Additionally, the *Lachnospira*, *Veillonella*, and *Dialister* populations within the guts of patients diagnosed with chronic kidney disease may exhibit a reduction in abundance when compared to healthy populations. However, the extent of this reduction may vary depending on the stage of the disease [107].

Researchers have demonstrated that a particular bacterial population may be more prevalent in individuals with chronic kidney disease. The correlation is illustrated in Table 4. S. T. Gryp et al. showed that an increased *Escherichia* population may occur in people with CKD [108]. Furthermore, a lower abundance of *Enterococcus* and *Klebsiella* could have been observed in people who do not suffer from chronic kidney disease [97,99,106]. Furthermore, H. Wang et al. observed that patients with chronic kidney disease exhibited an elevated prevalence of the *Flavonifractor* and *Citrobacter* genera [11]. These outcomes were also observed in other independent studies [104,105]. Nevertheless, an augmented prevalence of the Selenomonadaceae family as well as the *Akkermansia* genus has also been observed [11,106]. Additionally, the populations of *Desulfovibrio* and *Streptococcus* appeared to be diminished in healthy controls [103,109]. Additionally, an increased prevalence of *Oscillibacter* and *Alistipes* has been observed in individuals with chronic kidney disease, with a potential correlation between this elevation and the severity of the disease [107].

The scientific studies presented in Table 5 show that in patients with chronic kidney disease, the composition of the intestinal microbiota varies depending on the section of the gastrointestinal tract examined. Moreover, scientific researchers observed variations in the gut bacterial population in patients undergoing dialysis, including hemodialysis and peritoneal dialysis. In a study presented by G. P. Hobby et al., an elevated prevalence of bacteria belonging to the genera *Brachybacterium* and *Catenibacterium*, as well as the families Halomonadaceae and Pseudomonadaceae, was observed in dialysis patients [99]. Moreover, a potential decrease in the Prevotellaceae population was noted in patients with chronic kidney disease and those undergoing dialysis [97,99]. Furthermore, H. Wang et al. showed that in dialysis patients, the population of bacteria from the Proteobacteria phylum and the Enterobacteriaceae family could be increased, and the number of bacteria from the Euryarchaeota phylum and the Veillonellaceae, Lactobacillaceae, and Eubacteriaceae families could be decreased [11].

## 6. Potential Role of Probiotics in the Treatment of Chronic Kidney Disease

### 6.1. Gut Dysbiosis and Chronic Kidney Disease—Can We Treat Both?

Presumably, probiotics can alter gut microbiota in patients with chronic kidney disease (CKD). In CKD, the composition of gut microbiota often becomes imbalanced, leading to overgrowth of harmful bacteria and accumulation of uremic toxins (e.g., indoxyl sulfate, p-cresyl sulfate). The said imbalance, known as dysbiosis, promotes inflammation, oxidative stress, and eventually the progression of CKD [111].

Although small-scale studies and animal-based models have shown promising results, larger, well-controlled clinical trials are required to confirm the efficacy of probiotics in CKD management. The impact of probiotics on clinical outcomes like CKD progression and mortality in CKD and cardiovascular health patients remains an area of active research [112].

### 6.2. What Are Probiotics?

Probiotics are defined as live microorganisms that provide health benefits when consumed regularly in adequate amounts. Probiotics might be found in fermented foods such as yogurt and are also available as dietary supplements. Those microorganisms have the potential to support gut health by maintaining a healthy balance of gut microbiota, which influences various bodily functions, including the immune response, blood pressure, digestion, and even kidney function. Recent scientific research suggests the potential benefits of using probiotics in chronic kidney disease (CKD). The potential efficacy of probiotics in reducing uremic toxin levels and delaying CKD progression has been investigated in in vitro models, animal models, and CKD patients [113]. Moreover, synbiotics are a combination of both probiotics and prebiotics, which may facilitate the survival and implantation of live microbial food supplements in the gastrointestinal tract. The objective of synbiotics is to improve host health by enhancing the beneficial effects of probiotics and prebiotics [114]. Although the potential of probiotics and synbiotics in the treatment and prevention of diseases represents an exciting area of research, several challenges remain, as outlined by the authors in Table 6 [110].

### 6.3. Probiotics and Renal Failure: Mechanisms of Action

A review of scientific literature reveals a multitude of potential mechanisms through which probiotics may exert an effect on kidney failure. These include the following:

1. **Reduction in uremic toxins:** Probiotics could support a reduction in the production and absorption of uremic toxins by modifying the gut microbiota composition. Research has shown that specific probiotic strains, including Lactobacillus and Bifidobacterium, have the potential to decrease the levels of indoxyl sulfate and p-cresyl sulfate, which may potentially slow the progression of kidney disease [92].
2. **Improvement of gut barrier function:** Renal failure could compromise the integrity of the gut barrier, leading to increased intestinal permeability (leaky gut). Moreover, probiotics could strengthen the gut barrier by promoting the growth of beneficial bacteria that enhance gut integrity. These microorganisms might reduce the translocation of harmful bacteria and toxins from the gut into the bloodstream while also potentially mitigating systemic inflammation and further kidney damage [115].
3. **Metabolic benefits:** Probiotics have the potential to enhance metabolic profiles by increasing insulin sensitivity, reducing oxidative stress, and lowering lipid levels [21].
4. **Anti-inflammatory effects:** Chronic inflammation represents a pivotal element in the advancement of renal failure. Probiotics could exert anti-inflammatory effects by modulating the immune response and reducing the production of pro-inflammatory cytokines, thereby aiding in the protection of kidney tissues from further damage [116].
5. **Regulation of blood pressure:** Hypertension is both a cause and a consequence of renal failure. Some probiotic strains could help regulate blood pressure by producing bioactive peptides that inhibit angiotensin-converting enzyme (ACE), a key regulator of blood pressure. Improved blood pressure control can reduce the stress on the kidneys and slow the progression of kidney disease [117].

### 6.4. The Use of Probiotics and Synbiotics in Kidney Diseases

Several studies have yielded encouraging results in the treatment of renal diseases. A study published in the Journal of Renal Nutrition showed that patients with chronic kidney disease who received a probiotic supplement containing Lactobacillus and Bifidobacterium strains could exhibit a notable reduction in serum urea and creatinine levels compared to the control group. These findings suggest that probiotics may facilitate improvements in renal function and delay the necessity for dialysis [118]. Furthermore, research in patients with end-stage renal disease undergoing hemodialysis has indicated that probiotic supplementation may reduce inflammatory markers and improve quality of life. For example, a study published in the American Journal of Kidney Diseases reported that patients receiving a multi-strain probiotic exhibited decreased levels of C-reactive protein (CRP), a marker of inflammation [119]. Moreover, research in the field of acute kidney injury (AKI) remains scarce. However, animal studies have indicated that probiotics could reduce kidney injury by modulating the immune response and decreasing oxidative stress. These findings suggest potential benefits in the prevention of AKI in high-risk patients, such as those undergoing major surgery or experiencing severe infections.

### 6.5. The Use of Probiotics and Synbiotics in Chronic Kidney Disease

The scientific literature indicates that from the earliest stages of chronic kidney disease, there could be a quantitative and qualitative alteration of the intestinal microflora (dysbiosis), which could result in changes in the composition and metabolic activities of the microflora [120]. A review of the literature reveals that a multitude of observational, randomized, placebo-controlled, and double-blind studies have documented the beneficial effects of probiotics and synbiotics on CKD patients, even at the molecular level. The authors have compiled a comprehensive summary of these findings in Table 7 and Table 8.

## 7. Conclusions

The gut microbiota significantly impacts the development of various diseases, including chronic kidney disease. Extensive research has been conducted to investigate the relationship between the composition of the gut microbiota and the development of chronic kidney disease (CKD). It has been established that individuals with chronic kidney disease may exhibit dysbiosis in the composition of the intestinal microbiome, leading to the accumulation and accelerated translocation of uremic toxins due to compromised integrity of the intestinal epithelial barrier. This may result in an increase in oxidative stress and the onset of renal inflammation, which ultimately leads to renal damage. Patients with chronic kidney disease could derive benefit from dietary intervention incorporating prebiotics, probiotics, and synbiotics. This approach aims to modify the dysbiotic intestinal microbiome, which represents the primary goal in the treatment of uremic toxins in CKD. Furthermore, they have been shown to have a beneficial influence on the enhancement of epithelial barrier function and the regulation of blood pressure. The potential to alleviate symptoms and provide information about their impact on the pathogenesis and progression of chronic kidney disease is a source of considerable optimism.

It is important to note that there are outstanding unresolved issues: the short observation period, the lack of identification of the most suitable strains for intervention, and the variable individual response to probiotics. These factors make it impossible to reach a definitive conclusion on this matter now. Moreover, the significant individual variability in the intestinal microbiota of CKD patients, along with additional confounding factors such as diverse dietary patterns or other supplements, hinders the formation of objective conclusions. These challenges could enable researchers to ascertain the therapy’s full potential and contribute to groundbreaking results in the future. To improve the quality of future studies, it is important to define inclusion criteria related to patients’ diets and other supplements to eliminate any additional variables. In this review, the authors also propose to expand the study assessment to answer the question of whether dysbiosis within the microbiome occurs before or after laboratory manifestations of chronic kidney disease. If these changes manifest prior to the onset of CKD, we should monitor the microbiome in at-risk individuals to implement prevention earlier and modify patient-related risk factors before symptoms develop. Further research is imperative to evaluate the potential impact of introducing probiotic therapy, prebiotic therapy, or synbiotic therapy in delaying the onset of initial symptoms or progression of chronic kidney disease.

## Figures and Tables

**Table 1:** Categories of CKD based on GFR level [22].

Stages	GFR (mL/min/1.73 m^2^)	Classification
G1	>90	Normal or high
G2	60–89	Mildly decreased
G3a	45–59	Mildly to moderately decreased
G3b	30–44	Moderately to severely decreased
G4	15–29	Severely decreased
G5	<15	Kidney failure

GFR—glomerular filtration rate. CKD—chronic kidney disease.

**Table 2:** Categories of CKD based on albuminuria [22].

Category	AER (mg/24 h)	ACR (mg/mmol)	Classification
A1	<30	<3	Normal to mildly increased
A2	30–300	3–30	Moderately increased
A3	>300	>30	Severely increased

ACR—albumin-to-creatinine ratio. AER—albumin excretion ratio.

**Table 3:** Presentation of the lower quantity of bacterial populations in patients with chronic kidney disease in comparison to healthy individuals. The table presents the distribution of successive taxonomic levels, including cluster, class, order, family, genus and species, as well as Gram classification In consideration of the detail provided in the referenced works, when uncertainty existed regarding a specific taxonomic level, a cell in the table was left filled with the symbol ‘-’ to indicate that species was not known with certainty [11,97,102,103,104,105,106,107].

Phylum	Class	Order	Family	Genus	Species	Gram Classification	Presence in CKD
Bacteroidota	Bacteroidia	Bacteroidales	Bacteroidaceae	*Bacteroides*	*Fragilis*	Negative	Lower
Bacteroidetes	Bacteroidia	Bacteroidales	Prevotellaceae *	*Prevotella*	-	Negative	Lower
Bacillota	Bacilli	Lactobacillales	Lactobacillaceae *	*Lactobacillus*	-	Positive	Lower
Bacillota	Clostridia	Eubacteriales	Lachnospiraceae	*Roseburia*	*Hominis*	Positive	Lower
Bacillota	Clostridia	Eubacteriales	Oscillospiraceae	*Faecalibacterium*	*Prausnitzii*	Positive	Lower
Bacillota	Negativicutes	Vellionellales	Veillonellaceae *	*Veillonella*	*Parvula*	Negative	Lower
Bacillota	Clostridia	Eubacteriales	Lachnospiraceae	*Lachnospira*	-	Positive	Lower
Bacillota	Negativicutes	Veillonellales	Veillonellaceae *	*Dialister*	*Succinatiphilus*	Negative	Lower
Firmicutes	Clostridia	Clostridiales	Clostridiaceae	-	-	Positive	Lower
Bacillota	Clostridia	Clostridiales	Eubacteriaceae *	*Eubacterium*	*Rectale*	Positive	Lower
Firmicutes	Clostridia	Clostridiales	Ruminococcaceae	*Ruminococcus*	*Bromii*, *Callidus*	Positive	Lower
Bacillota	Clostridia	Eubacteriales	Lachnospiraceae	*Blautia*	-	Positive	Lower

* A lower presence of this family may also occur in patients undergoing dialysis in comparison to the healthy population [11,99].

**Table 4:** Presentation of the higher quantity of bacterial populations in patients with chronic kidney disease in comparison to healthy individuals. The table presents the distribution of successive taxonomic levels, including cluster, class, order, family, genus, and species, as well as Gram classification. In consideration of the detail provided in the referenced works, when uncertainty existed regarding a specific taxonomic level, a cell in the table was left filled with the symbol ‘-’ to indicate that the species was not known with certainty [11,97,99,103,104,105,106,107,108,109].

Phylum	Class	Order	Family	Genus	Species	Gram Classification	Presence in CKD
Firmicutes	Bacilli	Lactobacillales	Enterococcaceae *	*Enterococcus*	-	Positive	Higher
Pseudomonadota	Gammaproteobacteria	Enterobacteriales	Enterobacteriaceae *	*Klebsiella*	-	Negative	Higher
Bacillota	Bacilli	Lactobacillales	Streptococcaceae	*Streptococcus*	-	Positive	Higher
Pseudomonadota	Gammaproteobacteria	Enterobacterales	Enterobacteriaceae *	*Escherichia*	-	Negative	Higher
Proteobacteria *	Deltaproteobacteria	Desulfovibrionales	Desulfovibrionaceae	*Desulfovibrio*	-	Negative	Higher
Firmicutes	Clostridia	Clostridiales	Ruminococcaceae	*Oscillibacter*	-	Positive	Higher
Bacillota	Negativicutes	Selenomonadales	Selenomonadaceae	-	-	Negative	Higher
Bacillota	Clostridia	Eubacteriales	Oscillospiraceae	*Flavonifractor*	*Plautii*	Positive	Higher
Pseudomonadota	Gammaproteobacteria	Enterobacterales	Enterobacteriaceae *	*Citrobacter*	*Freundii*	Negative	Higher
Bacteroidota	Bacteroidia	Bacteroidales	Rikenellaceae	*Alistipes*	*Werkmanii*	Negative	Higher
Verrucomicrobiota	Verrucomicrobiae	Verrucomicrobiales	Akkermansiaceae	*Akkermansia*	-	Negative	Higher

* A higher presence of this family may also occur in patients undergoing dialysis in comparison to the healthy population [70,73].

**Table 5:** Presentation of the alterations in the composition of the gut microbiota in relation to the specific section of the digestive tract [110].

Intestinal Tract	Normal	ACKD/CKD
Stomach	*Helicobacter*, *Lactobacillus*	No change observed
Duodenum	*Lactococcus*, *Streptococcus*, *Staphylococcus*	Increased
Jejunum	*Streptococcus*, *Lactobacillus*, *Enterococcus*	Increased
Ileum	*Clostridium*, *Enterobacteriaceae*, *Bacteroides*	Increased
Colon	*Fusobacterium*	Aerobic overgrowth c.a. 100 times
	*Prevotellaceae*	*Bifidobacterium spp.*, *Lactobacillus*
	*Proteus*	*Acinetobcter*, *Proteus spp.*
	*Actinobacteria*	*Enterobacteria*, *E. coli*
	*Bacteroides*	*Proteobacteria*
	*Firmicutes*	Increased

CKD—chronic kidney disease. ACKD—advanced chronic kidney disease.

**Table 6:** Challenges and difficulties related to developing probiotic- and synbiotic-based therapy for chronic kidney disease [110].

Name of a Challenge	Importance
Strain-specific effects	Identifying a highly specific strain that could be useful and excluding those that are ineffective
Long-term safety and efficacy	Long-term studies are necessary to provide definitive evidence regarding the efficacy of the proposed treatment
Individual variability	The organism’s response to the probiotic is variable
Regulatory and quality issues	Ensuring the optimal quality and quantity of the product

**Table 7:** The impact of synbiotic usage on the gut microbiota in patients with chronic kidney disease.

Authors	Synbiotic	Results
T. Ogawa et al. [121]	*Bifidobacterium longum* JBL01 oligosaccharides	Decrease phosphorous levels that returned to baseline 2 weeks later
I. Nakabayashi et al. [122]	*Bifidobacterium breve* Yakult *Lactobacillus casei Shirota* galactooligosaccharides	Decrease in p-cresol in plasma Returning to correct bowel movement Connection of p-cresol level and constipation
J. Cruz-Mora et al. [123]	*Bifidobacterium lactis* *Lactobacillus acidophilus* inulin	Increase in *Bifidobacteria* in feces Decrease of *Lactobacilli* in feces Alleviation of gastrointestinal symptoms
D. Viramontes-Hörner et al. [124]	*Bifidobacterium lactis* *Lactobacillus acidophilus* Inulin	Diminishing of CRP and TNF-alpha levels. Alleviation of gastrointestinal symptoms
B. Guida et al. [125]	*Lactobacillus casei* subsp. *Rhamnosus*, *Lactobacillus plantarum*, *Bifidobacterium infantis*, *Lactobacillus gasseri*, *Lactobacillus salivarius*, *Streptococcus thermophilus*, *Lactobacillus sporogenes*, resistant tapioca starch and inulin	Decrease in p-cresol in plasma
M. Rossi et al. [126]	*Bifidobacteria* *Lactobacillus* *Streptococcus* Inulin Galactooligosaccharides Fructooligosaccharides	Increased *Bifidobacteria* Decreased Ruminococcaceae Slight increase in albuminuria No alteration in inflammation markers and oxidative stress Decrease PCS

**Table 8:** The impact of probiotic usage on the gut microbiota in patients with chronic kidney disease.

Authors	Probiotic	Results
M. L. Simenhoff et al. [127]	*Lactobacillus acidophilus*	↓ Dimethylamine, ↓Nitrosodimethylamine
F. Takayama et al. [128]	*Bifidobacterium longum* JCM008	↓ Indoxyl sulfate
K. Taki et al. [129]	*Bifidobacterium longum*	↓ Homocysteine, indoxyl sulfate, and triglycerides
Y. Ando et al. [130]	*Bifidobacterium longum*	Lowering of CKD’s progression in patients with con Cr ≥4 mg/dl or P ≥ 4 mg/dl
M. Hida et al. [131]	Lebenin	↓ p-cresol in feces and in serum
R. Natarajan et al. [132]	Renadyl	Reduction in CRP, leucocyte count, and indoxyl glucuronide
I.-K. Wang et al. [133]	*Bifidobacterium catenulatum* A302, *Lactobacillus plantarum* A87, *Bifidobacterium longum* A101, *Bifidobacterium bifidum* A218,	↑ IL-10 Slight preservation of kidney function ↓ TNF-α, IL-5, IL-6, and endotoxin
P. V. M. Alatriste et al. [110]	*Lactobacillus casei shirota*	↓ Urea
N. Ranganathan et al. [134]	*Streptococcus thermophilus* KB27, *Bifidobacterium longum* KB35, *Lactobacillus acidophilus* KB31	↑ Quality of life
N. Ranganathan et al. [135]	*Bifidobacterium longum* KB35, *Lactobacillus acidophilus* KB31, *Streptococcus thermophilus* KB27,	↑ Quality of life

↓—Decreased level of. ↑—Increased level of.

## Data Availability

The data used in this article is sourced from materials mentioned in the References section.
